# Discrimination of Smoke-Exposed Pinot Noir Wines by Volatile Phenols and Volatile Phenol-Glycosides

**DOI:** 10.3390/molecules30132719

**Published:** 2025-06-24

**Authors:** Armando Alcazar-Magana, Ruiwen Yang, Michael C. Qian, Yanping L. Qian

**Affiliations:** 1 Department of Food Science and Technology, Oregon State University, Corvallis, OR 97331, USA; armandoalkazar@gmail.com (A.A.-M.); maomaoyrw@163.com (R.Y.); michael.qian@oregonstate.edu (M.C.Q.); 2 College of Food Science and Engineering, Jilin University, Changchun 130062, China; 3 Department of Crop and Soil Science, Oregon State University, Corvallis, OR 97331, USA

**Keywords:** Pinot noir, smoke-exposure, smoke taint, volatile phenol, volatile phenol-glycoside, solid-phase microextraction and gas chromatography-mass spectrometry (SPME-GC-MS), high-performance liquid chromatography combined with high-resolution accurate Tandem mass spectrometry (HPLC–HR-MS/MS)

## Abstract

This study investigated the correlation between five primary volatile phenols (VPs) and their glycosides in smoke-exposed and non-smoke-exposed Pinot noir wines to assess and identify potential markers for smoke taint. The results showed that all putative VP-glycosides in smoke-exposed wines were higher than in non-smoke-exposed wines, with a fold change ranging from 2.11 to 31.88 for the top fifteen differentiations. VP-glycosides showed strong positive correlations among themselves, with correlation coefficients of 0.94 for hexose-guaiacol vs. pentose (P)-hexose (H)-cresol and 0.92 for syringyl-β-D-glucopyranoside vs. H-P-4-methylguaiacol. VP-glycosides also showed relatively high correlations with free and strong acid-hydrolyzed VPs. The correlation coefficient between H-P-guaiacol and free-form guaiacol is 0.71, and between H-P-guaiacol and total guaiacol is 0.78. The strong correlation suggests that these compounds are interconnected and regulated by the severity of smoke exposure. Multivariate analysis effectively differentiated smoke-exposed wines from non-smoke-exposed ones. However, more research is needed to fill the gaps in understanding smoke-derived compounds.

## 1. Introduction

From 2014 to 2023, an annual average of more than 60,000 wildfires burned over 7 million acres of land in the US, according to USAFACTS (https://usafacts.org/articles/how-much-damage-do-wildfires-do-in-the-us, accessed on 18 January 2025). Wildfire smoke impacts agricultural products by directly burning crops, orchards, and livestock, damaging infrastructure, and altering soil composition, sunlight, and ozone levels. It is challenging to determine the exact number of acres of grapes affected by wildfires each year due to the variability and difficulties in tracking specific crop impacts. However, in 2020, the haze from wildfires significantly damaged wine grapes, resulting in losses of USD 3.7 billion for the United States wine industry [1,2]. When vineyards are exposed to wildfire smoke, volatile phenols (VPs) and other smoke-related compounds can permeate the grape skins, potentially leading to “smoke taint” in wines, which can downgrade wine quality. The taint, described as smoky, burnt, dirty, ashy, etc., by sensory perception [3,4,5,6,7], is a significant concern for the wine industry.

Some VPs, including guaiacol, 4-methylguaiacol, 4-ethylguaiacol, 4-ethylphenol, syringol, *o*-, *p*-, and *m*-cresols, eugenol, vanillin, *cis*- and *trans*-oak lactone, furfural, thiophenols, etc., have been established to some extent as indicators of the smoke effect [6,8,9,10,11]. Although extensive research has been conducted to identify the volatile phenols contributing to smoke taint, the results are inconclusive because there are no universally accepted chemical markers or threshold levels for smoke taint. Additionally, the severity of smoke damage to wine grapes depends on several factors, including smoke intensity, fuel type, wind patterns, exposure time, grape variety, and microbial fermentation [8,12,13,14,15,16,17]. Some grape varieties absorb more of the offending taint compounds than others, while others are more tolerant [15]. Specifically, Syrah seems to favor a slightly smoky character. As a result, smoke taint is highly unpredictable, making it extremely challenging to determine which grapes to use and mitigate the risk of smoke taint [16].

Furthermore, volatile phenols can bind to sugar molecules to form stable glycosides [14,18,19,20]. Phenol glucosides, consistently found at elevated concentrations in smoke-affected wine, can function as smoke-taint precursors. They can be hydrolyzed to free VPs during winemaking and aging [21,22,23]. Thus, bounded-form precursors have also been evaluated as the potential smoke taint to improve the evaluation of smoke taint risk [5,6,24,25]. So far, all the glycosides identified in grapes or wine have the sugar directly bound to the aglycone as a β-D-glucose [26]. The second and third can be added to the β-D-glucose moiety to form disaccharide and trisaccharide glycosides. The tentatively identified phenolic glycosides, such as β-D-glucosyl-β-D-glucosides (gentiobiosides), β-D-glucopyranosides (monoglucosides), and disaccharides, have been found in smoke-affected grapes and wines at significantly elevated levels [16,18,27,28,29].

In 2020, a historic windstorm accompanied by hot, dry conditions triggered simultaneous “mega-fires” across Oregon, devastating wineries and other areas. The flavor chemistry laboratory at Oregon State University analyzed hundreds of wine samples for five primary volatile phenols (VPs), including guaiacol, 4-methylguaiacol, *o*-, *p*-, and *m*-cresols, to assess the potential for smoke taint, thereby enabling wineries to make informed decisions about harvest and wine production. This study aimed to characterize VP-glycosides in selected samples and correlate them with free and bound forms of VPs to help evaluate and identify potential markers for assessing smoke exposure.

## 2. Results and Discussions

### 2.1. Concentration of Free-Form and Total VPs in Non-Smoke-Exposed and Smoke-Exposed Wines

**Figure 1:**
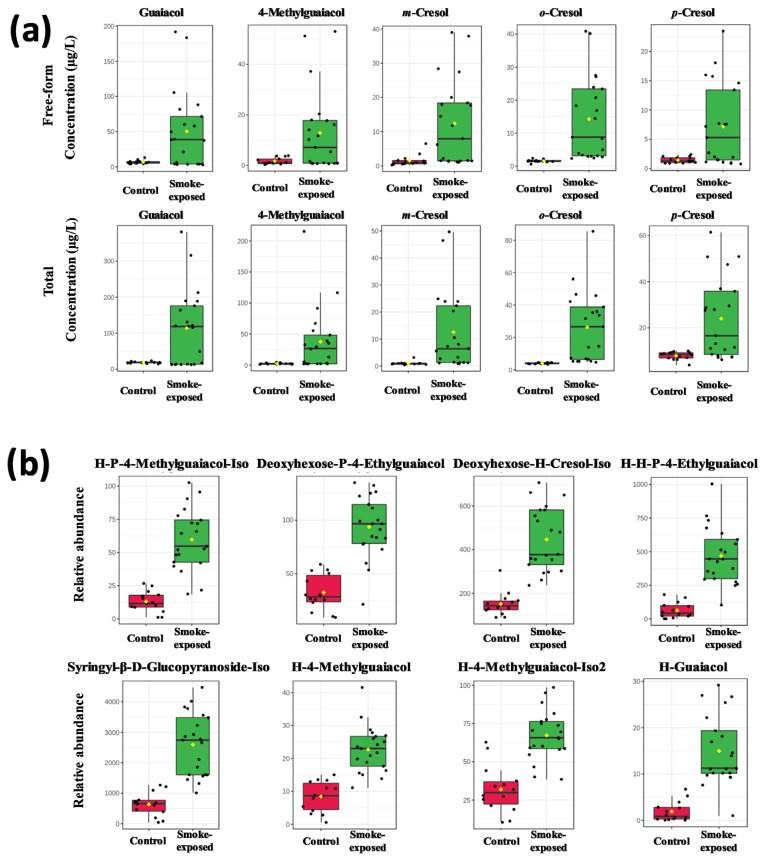
The bar chart of VPs and VP-glycosides for smoke-exposed and non-smoke-exposed wines (referred to as Control). Each dot corresponds to a sample. The yellow dot in each group corresponds to the mean. (**a**) Concentration of VPs (µg/L) was analyzed by SPME-GC-MS. (**b**) The relative abundance of VPs-glycosides was estimated from HPLC-HRMS/MS. H: hexose, P: pentose.

Quantification of free-form and total volatile phenols (VPs) is crucial for assessing the smoke-taint risk associated with smoke-impacted vintages. Calibration standard curves demonstrated good linearity (R^2^ > 0.9990) for all analytes (Table S1). The concentrations of the free and total VPs of the 14 non-smoke-exposed samples and 21 smoke-exposed ones were shown in Table S2 and Figure 1a. The free and total VPs concentration levels in the non-smoke-exposed wine provide baselines for the evaluation of smoky effect. The mean value of free-form guaiacol, 4-methylguaiacol, *o*-cresol, *p*-cresol, and *m*-cresol concentration for non-smoke-exposed wines was 6.8, 1.8, 1.5, 1.5, and 1.6 μg/L, respectively. Meanwhile, the mean value of total VPs concentration in non-smoke-exposed wines was 18.4, 2.3, 4.0, 7.8, and 1.0 μg/L, respectively, slightly higher than the reference baseline reported [30,31]. In comparison, for smoke-exposed wines, the mean concentrations of free-form guaiacol, 4-methylguaiacol, *o*-cresol, *p*-cresol, and *m*-cresol were 51.9, 13.3, 14.5, 7.4, and 12.7 μg/L, respectively, while the concentration of total VPs in smoke-exposed wines was 117.2, 38.9, 26.9, 24.4, and 12.8 μg/L, respectively.

As expected, results showed that the free-form and total VPs in smoke-exposed wines were higher than in non-smoke-exposed wines. The concentration of free-form guaiacol, 4-methylguaiacol, *o*-, *p*-, and *m*-cresol in smoke-exposed wine were 7.6, 7.5, 9.4, 5.1, and 8.4-fold, respectively, higher than that of non-smoke-exposed wines. Whereas total guaiacol, 4-methylguaiacol, *o*-, *p*-, and *m*-cresol concentrations in smoke-exposed wine were 6.4, 17.0, 6.8, 3.1, and 13.3-fold of those in non-smoke-exposed wine, respectively. Although VPs have been identified as potential marker compounds, it has not been unequivocally demonstrated that they are the root cause of wine smoke taint. The contribution of VPs to smoke taint has been discussed in our previous publication, and free-form cresols have been identified as significant factors by statistical analyses [25].

### 2.2. VP-Glycoside Analysis in Wines by HPLC–HR-MS/MS

Glycosides in non-smoke-exposed and smoke-exposed wine samples were characterized by HPLC–HRMS/MS. As reported, level 2 annotations (putatively annotated metabolites) were produced by querying and matching MS/MS spectral data with open-access databases [32]. Additionally, glycosides reported in the literature for smoked taint wine were included in the study by matching exact mass and MS/MS data [33]. Despite the high-throughput analysis provided by HPLC–HRMS/MS, including elemental composition, MS/MS data, and isotopic patterns, it is challenging to assign the order of sugars and their absolute configuration when analyzing disaccharides. To avoid the possibility of false positives, when isomers were present, the compounds were indicated as such, and all the annotations were considered putative (level 2, L2). From this search, 35 different VP-glycosides were tentatively annotated according to the criteria established by the Metabolomics Standards Initiative [34,35]. Detailed information about VP-glycosides, including accepted compound identification, adducts detected, exact mass, mass error, and formula, were listed in Table S3. The top eight most discriminant glycosides identified in this study were hexose (H)-pentose (P)-4-methylguaiacol-Iso, deoxyhexose-P-4-ethylguaiacol, deoxyhexose-H-cresol-Iso, H-H-P-4-ethylguaiacol, syringyl-β-D-glucopyranoside-Iso, H-4-methylguaiacol, H-4-methylguaiacol-Iso2, and H-guaiacol (Figure 1b). These results were in agreement with literature that disaccharides are the dominant VP-glycosides in smoke-exposed grapes and wines [18]. H-P-glycosides are consistently identified as the most abundant VP-glycosides in smoke-exposed *V. vinifera* [24], among others, including syringyl-β-D-glucopyranoside, guaiacyl-β-D-gentiobioside, and syringyl-β-D-gentiobioside. Deoxyhexose-H-cresol, deoxyhexose-H-P-phenol, deoxyhexose-P-4-ethylguaiacol, H-guaiacol, H-H-P-4-ethylguaiacol, H-P-4-methylguaiacol, H-P-guaiacol, H-P-P-4-methylguaiacol, P-H-cresol, and P-P-H-cresol have been identified in smoke-exposed grapes by HPLC-qTOF-MS [33].

### 2.3. Heating Map Analysis of VPs and Glycosides for Smoke-Exposed and Non-Smoke-Exposed Wines

**Figure 2:**
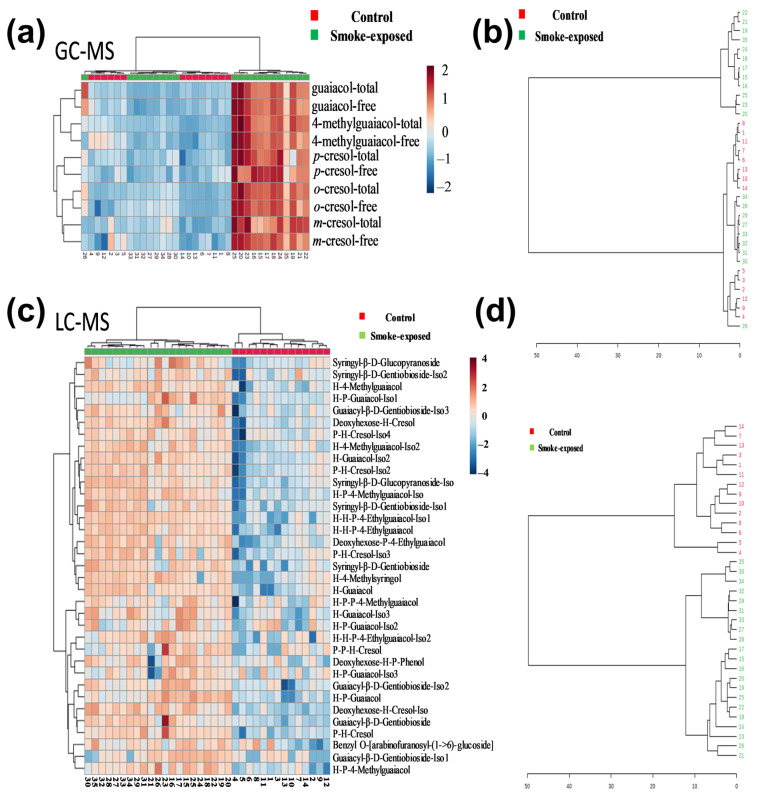
Unsupervised analysis for VPs and VP-glycosides. Hierarchical clustering heatmaps (**a**,**c**) and dendrograms (**b**,**d**) were constructed based on VPs concentration (µg/L) and the relative abundance of VP-glycosides, respectively. Each colored cell on the map corresponds to a wine sample. Samples 1–14 correspond to non-smoke-exposed wines (Control), while 15–35 correspond to smoke-exposed wines.

To visualize the differences between the non-smoke-exposed and smoke-exposed wines, a hierarchical clustering heatmap and dendrogram of VPs concentration across wine samples were performed using the online software MetaboAnalysis V5.0 (Figure 2). Each color-coded cell on the heatmap corresponded to a measurement. VP compounds were displayed in columns, and wine samples were arranged in rows (Figure 2a). Figure 2a showed that the wine samples were well separated into two distinct groups. A few smoke-exposed wines, samples 27 to 34, had similar VP concentrations to the non-smoke-exposed samples, which were categorized in the same manner, as demonstrated by the cluster dendrogram (Figure 2b, Table S2). As stated earlier, the severity of smoke damage to wine grapes depends on factors such as exposure duration and location. However, the glycosides of samples 27 to 34 were categorized with the rest of the smoke-exposed samples when the dendrogram was performed using VP-glucosides, implying that VP-glycosides may have more discrimination power than VPs alone. Hierarchical clustering heatmaps and dendrograms computed using the relative abundance of the 35 tentatively identified VP-glycosides in non-smoke-exposed and smoke-exposed wines support the idea of using VP-glycosides as potential markers of smoke exposure (Figure 2c,d).

### 2.4. PCA Analysis of VPs and VP-Glycosides

**Figure 3:**
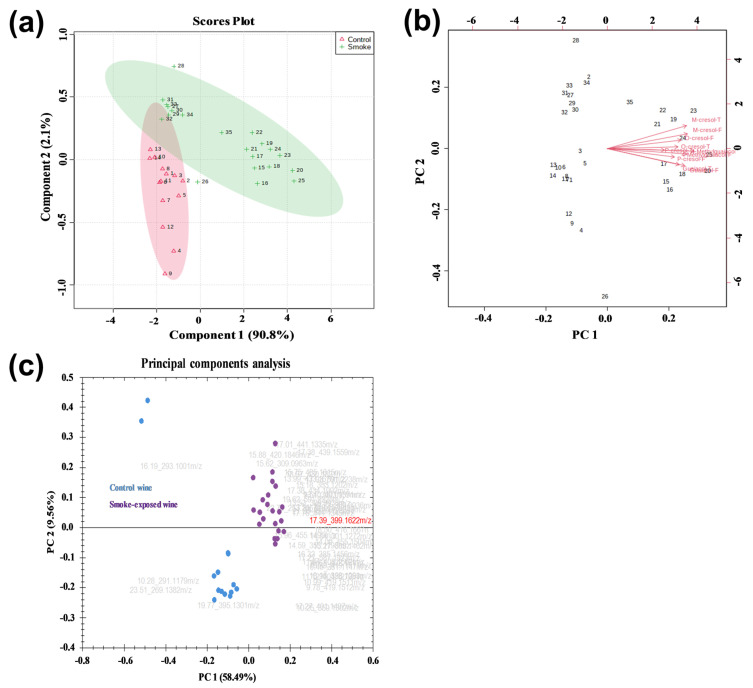
Unsupervised principal component analysis (PCA) for VPs and VP-glycosides. (**a**) Score plot-PCA for free VPs in non-smoke-exposed and smoke-exposed wines (Control). Explained variances are shown in the corresponding axes. (**b**) Biplot between the two selected PCs. Each dot corresponds to a wine sample. Sample numbers 1 to 14 correspond to non-smoke-exposed wines (Control), while 15 to 35 correspond to smoke-exposed wines. (**c**) Unsupervised principal component analysis for VP-glycosides. Each dot corresponds to a wine sample. PC1 and PCA2 describe 68% of the variation among samples. PCA was computed using Progenesis QI (version 2.3).

PCA is an unsupervised method that expresses the variance of a multidimensional dataset with fewer dimensions using principal components (PCs). As a result, the discriminant variables of each group can be distinguished, and the relationship between samples can be established [36]. Here, PCA was used to visualize the dissimilarities between non-smoke-exposed and smoke-exposed wines. As shown in Figure 3a, the two principal components accounted for 90.8% (PC1) and 2.4% (PC2) of the total variance. The cumulative contribution was much higher than the 60% mark, which is generally considered a good separation model [37]. Thus, good separation was observed for the two clusters in the present study. The smoke-exposed wines exhibited greater data dispersion, probably due to different degrees of exposure to wildfire smoke. In agreement with the dendrogram, samples 27–34 were closely related to non-smoke-exposed wines yet separated well from them. A biplot was performed (Figure 3b) to facilitate visualization of the relationship between loadings (samples) and scores (VPs).

PCA analysis was also performed to capture the similarities and differences between the 35 tentative annotated VP-glycosides (Figure 3c). Two principal components accounted for close to 70% of the total variance of the data (58.49% and 9.56% for PC1 and PC2, respectively). The distinct separation of the two categories suggested that VP-glycosides could be potential biomarkers of smoke exposure.

Univariate analysis identified the top 15 volatile phenol glycosides to differentiate non-smoke-exposed and smoke-exposed wines. The *p*-values of the top 15 VP-glycosides were very low (*p* < 9 × 10^−5^, Table 1), with a fold change (FC) ranging from 2.11 to 31.88, indicating that the contents of the top 15 VP-glycosides were significantly affected by smoke. The higher the FC number, the greater the difference between the smoke-exposed and non-smoke-exposed samples.

### 2.5. Correlation of Glycosides to the Free and Total Form of VPs

**Figure 4:**
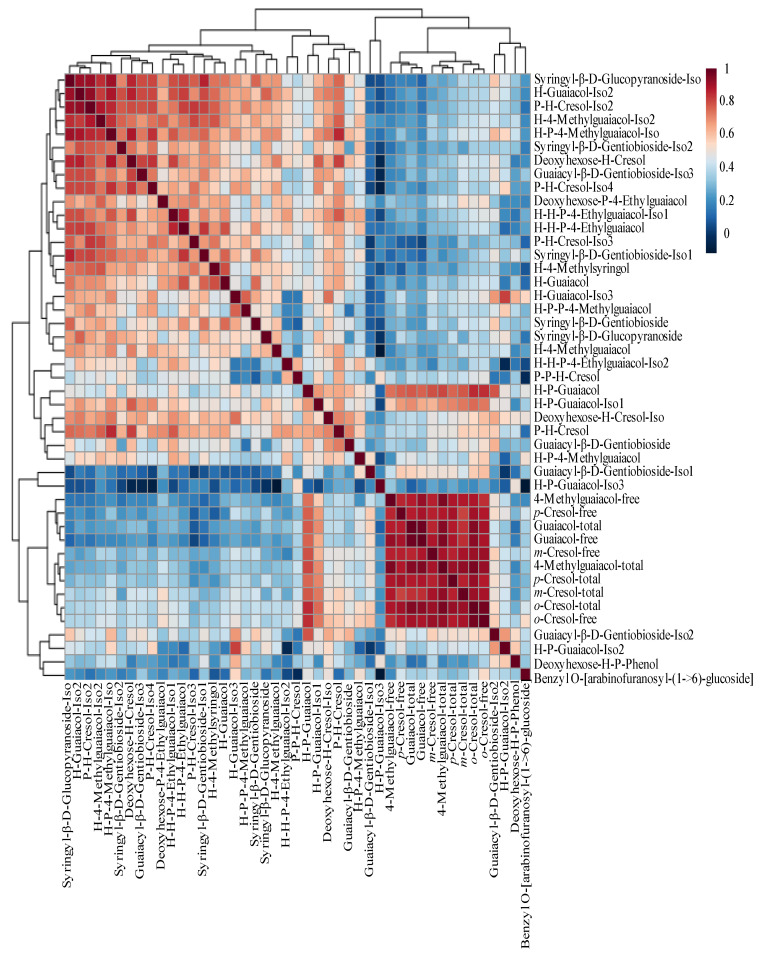
The heatmap of 35 tentatively annotated VP-glycosides with VPs, as determined by Pearson’s correlation analysis. The darker the red color, the stronger the correlation. All correlations were positive. H—hexose, P—pentose.

Correlation analysis was performed to evaluate potential connections between VPs and putative VP-glycosides. The correlation heatmap and matrix for 35 tentative annotated VP-glycosides and the VPs (free-form and total) were plotted using Pearson’s correlation analysis (Figure 4). The correlation matrix identifies positive or negative correlations between different compounds—values range from 1 (darkest red) to −1 (darkest blue). The results showed that VPs and VP-glycosides were all positively correlated (Table S4), with nearly 60% of the correlations being statistically significant (*p* < 0.05). For example, H-P-guaiacol vs. free-form guaiacol (r = 0.77, *p* = 2.50 × 10^−8^), H-P-guaiacol vs. total guaiacol (r = 0.78, *p* = 6.60 × 10^−8^), H-P-guaiacol-iso1 vs. free-form guaiacol (r = 0.65, *p* = 2.36 × 10^−5^), and H-P-guaiacol-iso1 vs. total guaiacol (r = 0.71, *p* = 2.19 × 10^−6^) showed the strong correlation in agreement with the findings [18]. The positive correlations indicated the potential interaction among their biosynthetic pathways, converting the VP to VP-glycosides.

However, the closest relationships were among free-form and bound-form VPs, as indicated by the dark red square at the bottom right of the heat map (Figure 4). In addition, strong correlations were also observed between the VP-glycosides, such as syringyl-β-D-glucopyranoside vs. H-guaiacol (r = 0.92), syringyl-β-D-glucopyranoside vs. P-H-cresol (r = 0.92), syringyl-β-D-glucopyranoside vs. H-P-4-methylguaiacol (r = 0.92), H-guaiacol vs. P-H-cresol (r = 0.94), and H-guaiacol vs. H-P-4-methylguaiacol (r = 0.90).

## 3. Materials and Methods

### 3.1. Materials

#### 3.1.1. Wine Samples

Fourteen non-smoke-exposed (referred to as “control” in bar charts and figures due to space limitation) Pinot noir wine samples (numbered 1 to 14) were randomly selected from Oregon State University research vintages made between 2013 and 2016 without barrel aging. They were stored at −20 °C before analysis. Twenty-one smoke-exposed wines with a risk of smoke taint (numbered 15 to 35) were obtained from grape growers and winemakers in the summer of 2020, the most destructive wildfire season on record in Oregon, and analyzed promptly.

#### 3.1.2. Chemicals and Reagents

Standards of guaiacol (≥98%), 4-methylguaiacol (99%), *o*-cresol (≥99.5%), *p*-cresol (≥99%), and *m*-cresol (99%) were obtained from Sigma-Aldrich (St. Louis, MO, USA). 2-Methoxyphenol-3, 4, 5, 6-*d*_4_ (guaiacol-*d*_4_; 98.5%), 2-methoxy-4-methylphenol-3, 5, 6-*d*_3_ (4-methylguaiacol-*d*_3_; 99.3%), *m*-cresol-*d*_7_ (98.7%), *p*-cresol-*d*_7_ (99.2%), and *o*-cresol-*d*_7_ (99%) were purchased from CDN Isotopes (Pointe-Claire, QC, Canada). Tartaric acid (AR grade) was purchased from Johnson Matthey Company (Heysham, Lancs, UK). Ethanol (HPLC grade) was purchased from Greenfield Global USA Inc. (Brookfield, CT, USA). Methanol (HPLC and LC-MS grade) and sodium hydroxide 10 N solution (30% *w*/*w*) were purchased from Fisher Scientific (Fair Lawn, NJ, USA). Citric acid, sodium chloride (ACS grade), and hydrochloric acid were purchased from EMD Millipore Corporation (Billerica, MA, USA). Milli-Q water was obtained from a Milli-Q purification system (Millipore, Boston, MA, USA).

Synthetic wine was used to establish standard curves for quantifying volatiles. It was prepared by dissolving 3.5 g of tartaric acid in one liter of 12% aqueous ethanol and adjusting the pH to 3.2 with sodium hydroxide [38].

### 3.2. Quantification of Free-Form VPs in Wines

#### 3.2.1. Internal Standard (IS) Preparation

Five volatile phenols, including guaiacol, 4-methylguaiacol, *m*-cresol, *o*-cresol, and *p*-cresol, were quantified in wine samples. Stable isotope-labeled compounds were used as internal standards (IS). The IS (guaiacol-*d*_4_, 4-methylguaiacol-*d*_3_, *o*-cresol-*d*_7_, *p*-cresol-*d*_7_, and *m*-cresol-*d*_7_) were dissolved in methanol and then mixed to get a final concentration of 10 mg/L (ppm).

#### 3.2.2. Wine Sample Preparation

Two milliliters of wine sample were diluted with 8 mL citric acid buffer saturated with salt (0.2 M, pH 3.5) in a 20 mL auto-sampler glass vial. Then, 10 μL of a mixture of IS was added, and the cap was tightened. A duplicate sample and a quality control standard were run every ten samples.

#### 3.2.3. Standard Curve Preparation

Individual standard compounds of guaiacol, 4-methylguaiacol, *o*-cresol, *p*-cresol, and *m*-cresol were weighed and dissolved separately in 1 mL of methanol. Then, a mixture of stock solution (500 mg/L) was obtained by mixing the above individual standard compounds. Nine data points with an incremental concentration of the sub-stock standard solution (0.01–5 mg/L) were prepared by diluting the stock solution with pure methanol. All stock solutions were frozen until use.

A linear calibration curve ranging from 0.05 to 25 µg/L of analytes was prepared in a 20 mL sample vial by mixing 50 µL of a mixture of sub-stock standards, 10 µL of internal standard (IS), 2 mL of synthetic wine, and 8 mL of citric acid buffer solution (0.2 M, pH 3.5).

#### 3.2.4. Free-Form VPs Quantification by Gas Chromatography-Mass Spectrometry (GC-MS)

Quantification of free-form VPs was analyzed using an Agilent 6890 gas chromatograph equipped with an Agilent 5973 mass selective detector (Agilent Technologies, Inc., Santa Clara, CA, USA). The free-form VPs in the samples were extracted using a 50/30 μm Divinylbenzene/Carboxen/Polydimethylsiloxane (DVB/CAR/PDMS) fiber (Supelco Inc., Bellefonte, PA, USA) at 50 °C for 25 min with stirring at 250 rpm. The extraction was handled by a multipurpose autosampler (Gerstel Inc., Linthicum, MD, USA). Then, the volatiles were desorbed in the GC port at 250 °C for seven minutes in splitless mode. A SUPEL COWAX^TM^-10 column (59 m × 250 μm × 0.25 mm film, Supelco Inc.) was used for the separation. The flow rate of the carrier gas (helium) was 2 mL/min. The oven temperature was initially set at 60 °C for 3 min, then ramped to 160 °C at a 20 °C/min rate, to 200 °C at a 5 °C/min rate, and held for 10 min. Finally, it was ramped to 230 °C at a rate of 20 °C/min and held for 5 min. The transfer line and MS source temperatures were 280 °C and 230 °C, respectively. Selective ion monitoring (SIM) mode was adopted to acquire the data. Selected ions were used to build the calibration curve using ChemStation software (Version D.03.00.552) (Table S1, as Supplemental Information).

### 3.3. Quantification of Total VPs in Wines

Total VPs were analyzed after acid hydrolysis, as described by Noestheden et al. (2017), with modifications [10]. In brief, 2 mL of wine samples and 20 μL of 10 M HCL were mixed in 20 mL headspace glass vials to achieve a pH of 1.2. The vials were held in a water bath (100 °C) for four hrs. After cooling with ice, 8 mL of citric acid buffer (0.2 M, pH 3.5) saturated with NaCl and 20 μL of IS (10 mg/L) were added to the wine sample. A duplicate was prepared for every ten samples. Total VPs were analyzed on an Agilent 7890A gas chromatograph equipped with an Agilent 5975C MSD (Agilent Technologies, Inc.). Compounds were separated on an OPTIMA^®^ FFAP plus column (60 m × 250 μm × 0.5 mm, Macherey-Nagel Inc., Düren, Germany). Other instrument settings for total VPs analysis were the same as previously described.

### 3.4. Untargeted Data-Dependent Acquisition Analysis of VP-Glycosides by HPLC-HR-MS/MS

Untargeted VP-glycoside analysis in wine was conducted according to Alcazar Magana et al. (2020) [32] with some modifications. A Shimadzu (Tokyo, Japan) Nexera UHPLC system, connected to a high-resolution AB SCIEX Triplet OF 5600 (SCIEX, Framingham, MA, USA) mass spectrometer equipped with a Turbo V ionization source, was operated in positive electrospray ionization (ESI) mode. Chromatographic separation was accomplished using an Inertsil Phenyl-3 column (4.6 mm × 150 mm, 100 Å, five μm; GL Sciences, Torrance, CA, USA). One milliliter of each wine was diluted five times with aqueous methanol (70% *v*/*v*) and then centrifuged at 10,000 rpm for 5 min. From the supernatant, 5 μL was injected in triplicates. Gradient elution was performed using a mobile phase consisting of solvent A (water containing 0.1% *v*/*v* formic acid) and solvent B (methanol containing 0.1% *v*/*v* formic acid). The flow rate was 0.4 mL/min. The chromatographic method was 30 min, and the gradient was as follows: an initial one min at 5% B, followed by 5 to 30% B from 1 to 10 min, then 30 to 100% B from 10 to 20 min, hold at 100% B from 20 to 25 min, and then return to 5% B from 25 to 30 min.

Data-dependent acquisitions (DDAs) were performed to obtain precursor and fragment ion information, which facilitated the annotation of compounds in the wine samples. DDA analyses were performed using positive ionization mode (ESI+). The following parameter settings were used for detecting positive ions: spray voltage of 4500 V, source temperature of 550 °C, and a period cycle time of 950 ms. In addition, the following settings were used: full scan with ion accumulation of 150 ms, followed by a dynamic MS/MS selection of the eight most intense ions with 100 ms accumulation; after two MS/MS acquisitions, the precursor (fragmented) ions were excluded for 30 s; collision energy 35 V with collision energy spread (CES) of 15 V ramped through each MS/MS scan using a range of *m*/*z* 100–1200. Mass calibration was automatically performed after every two hours with a calibrant delivery system.

### 3.5. Data Processing and Annotation of Wine Metabolites

**Figure 5:**
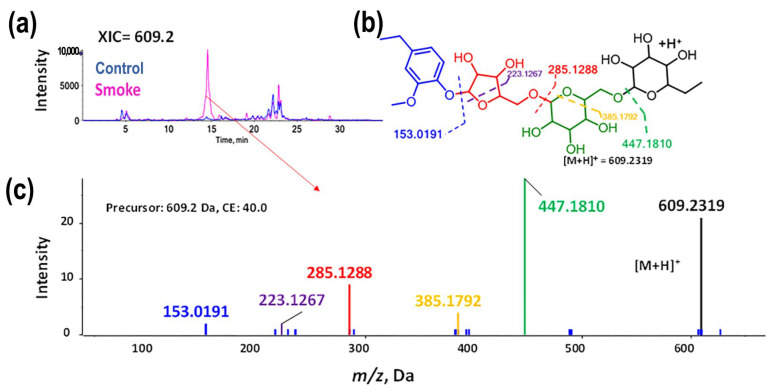
The fragmentation pathway (color-coded) is an example of using the MS/MS data for level 2 annotations. (**a**) Extracted ion chromatogram *m*/*z* 609.2. (**b**) Proposed fragmentation pattern for Hexose-Hexose-Pentose-4-Ethylguaiacol; the dotted lines represent the sequential fragmentation. (**c**) Spectra acquired for *m*/*z* 609.2 using a collision energy of 40 V.

Annotation confidence was established according to reporting criteria for chemical analysis suggested by the Metabolomics Standards Initiative [34,35]. The level 2 (L2, considered the cut-off; any sample’s value above the cut-off is regarded as a positive result; below the cut-off is considered negative) annotations were based on exact mass, isotopic pattern, and MS/MS spectral data (Figure 5). Also, two manual data evaluations were included: (1) examination of the metabolite structures concerning the suitability of the ionization mode in which a compound was detected, and (2) elution peaks for tentatively annotated features were interrogated to omit compounds originating from in-source fragmentation.

### 3.6. Statistical Analysis

Data distribution, correlation matrix, hierarchical clustering heatmaps, hierarchical clustering dendrogram, principal component analysis (PCA), and Pearson correlation analysis were conducted using an online version of MetaboAnalyst V5.0 (www.metaboanalyst.ca, Xia Lab at McGill University, Montreal, Canada, accessed on 8 May 2024). In addition, hierarchical cluster analysis was performed using Euclidean distance with Ward’s linkage algorithm [39].

## 4. Summary

The free form and total volatile phenols and their glycosides in smoke-exposed and non-smoke-exposed Pinot noir were analyzed by SPME-GC-MS and HPLC-HR-MS/MS. Results showed that smoke exposure significantly increases the concentration of VPs and glycosides in wine, which is well separated from non-smoke exposure wines by a heating map and PCA analysis. Moreover, fifteen VP-glycosides identified were much higher in smoke-exposed wines, strongly correlated with each other and with free and total VPs, suggesting a coordinated regulation of these compounds in response to smoke exposure. The findings inform future efforts to mitigate the adverse effects of smoke exposure on wine quality. However, further structural characterization is advised to ensure a complete assessment of the presence of VP-glycosides.

## Figures and Tables

**Table 1:** Univariate fold change analysis of top 15 volatile phenol glycosides identified from non-smoke-exposed and smoke-exposed wines.

	Tentative Glycosides	Fold-Change (FC)	Raw *p*-Value
1	H-P-4-Methylguaiacol-Iso	4.58	2.37 × 10^−8^
2	Deoxyhexose-P-4-Ethylguaiacol	2.88	2.59 × 10^−8^
3	Deoxyhexose-H-Cresol-Iso	2.97	3.02 × 10^−8^
4	H-H-P-4-Ethylguaiacol	7.39	6.61 × 10^−8^
5	Syringyl-β-D-Glucopyranoside-Iso	4.08	9.50 × 10^−8^
6	H-4-Methylguaiacol	2.69	1.26 × 10^−7^
7	H-4-Methylguaiacol-Iso2	2.11	2.52 × 10^−7^
8	H-Guaiacol	7.88	3.95 × 10^−7^
9	P-H-Cresol-Iso2	3.90	5.13 × 10^−7^
10	Syringyl-β-D-Gentiobioside-Iso1	2.60	8.96 × 10^−7^
11	H-H-P-4-Ethylguaiacol-Iso1	31.88	2.21 × 10^−5^
12	P-H-Cresol-Iso3	3.52	3.23 × 10^−5^
13	H-P-4-Methylguaiacol	2.66	4.37 × 10^−5^
14	Deoxyhexose-H-Cresol	3.13	5.73 × 10^−5^
15	Syringyl-β-D-Gentiobioside	5.65	8.64 × 10^−5^

Abbreviations: H—Hexose, P—Pentose, Iso—Isomer.

## Data Availability

The original contributions presented in this study are included in the article. Further inquiries can be directed to the corresponding author.

## Supplementary Materials

The following supporting information can be downloaded at: https://www.mdpi.com/article/10.3390/molecules30132719/s1, Table S1. Standard curves for free and total volatile phenol analysis by HS-SPME-GC-MS. Table S2. Concentrations of volatile phenols analyzed by SPME-GC-MS. Table S3. Thirty-five tentative annotated glycosides (level 2 annotations) identified from non-smoke-exposed and smoke-exposed wines. Table S4. Correlation matrix for VPs and VP-glycosides in non-smoke-exposed and smoke-exposed wines with Pearson’s correlation analysis.

## Abbreviations

VPs	Volatile phenols
SPME-GC-MS	Solid-phase microextraction and gas chromatography-mass spectrometry
HPLC–HR-MS/MS	High-performance liquid chromatography combined with high-resolution accurate Tandem mass spectrometry
2D-COS	Two-dimensional correlation spectroscopy
MIR	Mid-infrared
SIDA	Isotope dilution assay
AWRI	Australian Wine Research Institute’s Commercial Services Laboratory
SBSE	Stir bar sorptive extraction
uHPLC-QToF	Ultrahigh-performance liquid chromatography-quadrupole time-of-flight mass spectrometry
IS	Internal standards
MSD	Mass selective detector
SIM	Selective ion monitoring
ESI	Electrospray ionization
DDAs	Data-dependent acquisitions
CES	Collision energy spread
PCA	Principal component analysis
PLS-DA	Partial least squares-discriminant analysis
