# Supplementary material for: Discrimination of Smoke-Exposed Pinot Noir Wines by Volatile Phenols and Volatile Phenol-Glycosides
Source: Molecules. 2025 Jun 24;30(13):2719. doi: 10.3390/molecules30132719 (PMC12250721; doi:10.3390/molecules30132719)
Supplement: Supplementary file 1 [file molecules-30-02719-s001.zip › molecules-3676364-supplementary.pdf]

Discrimination of Smoke-Exposed Pinot Noir Wines by Volatile Phenols and Volatile Phenol-Glycosides

Armando Alcazar-Magana [1], Ruiwen Yang [1,2], Michael C. Qian [1] and Yanping L. Qian [3,*]

[1] Department of Food Science and Technology, Oregon State University, Corvallis, OR 97331, USA; armandoalkazar@gmail.com (A.A.-M.); maomaoyrw@163.com (R.Y.); michael.qian@oregonstate.edu (M.C.Q.)

[2] College of Food Science and Engineering, Jilin University, Changchun 130062, China

[3] Department of Crop and Soil Science, Oregon State University, Corvallis, OR 97331, USA

* Correspondence: yan.ping.qian@oregonstate.edu; Tel.: +1-541-737-1135

Table S1. Standard curves for free and total volatile phenol analysis by HS-SPME-GC-MS

Compounds	*m/z*	Linear range (μg/L)	R^2		Equation	
			Free-form	Total	Free-form	Total
guaiacol-d_4 (IS)	113, 128					
guaiacol	124, 109	0.05-25	0.9999	0.9999	y=0.8874x+0.02406	y=0.9369x+0.0038
4-methylguaiacol-d_3 (IS)	141, 126					
4-methylguaiacol	138, 123	0.05-25	0.9998	0.9999	y=0.8885x+0.03685	y=0.9299x+0.0068
o-cresol-d_7 (IS)	115, 113					
o-cresol	108, 107	0.05-25	0.9997	0.9999	y=1.035x+0.02836	y=1.032x-0.00085
p-cresol-d_7 (IS)	115, 113					
p-cresol	108, 107	0.05-25	0.9996	0.9999	y=1.205x+0.06058	y=1.170x+0.0020
m-cresol-d_7 (IS)	115,113					
m-cresol	108, 107	0.05-25	0.9996	0.9997	y=1.428x+0.06449	y=1.454x+0.0015

IS: Internal Standard

Table S2. Concentrations of volatile phenols analyzed by SPME-GC-MS.

		**Total form (free + bound) VPs (μg/L)**					**Free-form VPs (μg/L)**				
**Sample number**	**Smoke exposure**	**guaiacol**	**4-methylguaiacol**	***o*-cresol**	***p*-cresol**	***m*-cresol**	**guaiacol**	**4-methylguaiacol**	***o*-cresol**	***p*-cresol**	***m*-cresol**
1	Non-smoke	18.0	1.8	4.4	8.5	1.0	7.5	0.9	1.8	2.1	0.8
2	Non-smoke	20.3	3.2	4.6	9.9	3.2	7.6	2.7	1.3	1.0	6.5
3	Non-smoke	17.8	2.2	4.4	9.8	1.080	6.6	1.6	2.2	2.4	2.2
4	Non-smoke	23.4	3.9	4.2	7.0	0.8	13.1	3.9	1.6	1.6	1.0
5	Non-smoke	22.0	2.4	4.2	8.8	0.9	10.5	2.2	2.4	1.9	3.5
6	Non-smoke	15.2	1.6	3.6	7.4	0.55	5.3	1.0	1.4	1.3	1.4
7	Non-smoke	18.7	1.8	3.4	8.2	0.6	7.2	1.3	1.4	1.1	1.6
8	Non-smoke	18.7	1.6	4.0	6.2	0.7	6.2	0.7	1.7	2.1	1.2
9	Non-smoke	19.0	3.6	3.8	9.1	1.1	8.3	3.8	0.6	1.3	0.5
10	Non-smoke	18.6	1.2	3.9	6.2	0.9	5.4	0.6	1.4	1.0	0.5
11	Non-smoke	16.5	2.0	3.8	9.3	1.0	4.3	1.1	1.4	1.9	0.5
12	Non-smoke	17.2	4.4	3.9	9.2	0.8	5.1	3.7	1.4	0.9	0.2
13	Non-smoke	14.4	1.2	3.6	6.8	0.5	4.4	0.5	1.4	0.9	0.7
14	Non-smoke	17.8	1.3	3.6	3.8	0.5	4.4	0.6	1.6	0.9	0.6
**Mean**	**Non-smoke**	**18.4**	**2.3**	**4.0**	**7.8**	**1.0**	**6.8**	**1.8**	**1.5**	**1.5**	**1.6**
15	Smoke	121.1	29.3	35.3	27.9	5.6	58.1	14.1	20.8	14.6	17.9

16	Smoke	130.8	32.6	36.0	36.9	6.4	60.3	16.2	16.8	18.1	18.4
17	Smoke	120.0	26.2	33.6	29.5	8.0	49.8	11.8	18.3	13.4	14.4
18	Smoke	189.6	48.4	46.6	35.8	10.5	88.2	20.4	27.1	15.8	27.4
19	Smoke	188.7	67.1	38.8	11.0	23.9	81.8	17.82	23.9	5.3	20.0
20	Smoke	381.2	215.4	85.5	61.5	23.9	183.5	51.2	40.9	7.2	39.0
21	Smoke	119.0	38.9	26.6	28.7	22.3	37.6	7.2	8.4	7.5	8.0
22	Smoke	115.8	35.2	31.6	27.6	20.4	39.2	10.2	14.4	7.6	11.8
23	Smoke	212.5	91.5	45.8	47.5	46. 5	105.6	37.2	27.5	7.7	28.4
24	Smoke	164.0	55.4	42.1	50.8	24.9	71.6	18.0	23.4	16.0	17.7
25	Smoke	316.0	116.6	56.0	50.9	49.7	191.9	53.0	40.2	23.5	37.9
26	Smoke	176.0	5.2	13.9	13.1	0.8	38.6	0.9	4.9	2.0	1.4
27	Smoke	13.2	1.8	5.2	7.4	1.3	4.0	1.0	3.3	1.1	1.3
28	Smoke	13.7	2.0	7.2	10.5	3.2	3.7	0.8	3.1	0.9	1.6
29	Smoke	12.6	2.2	5.3	8.4	1.2	4.0	0.9	2.8	1.5	1.461
30	Smoke	12.7	2.1	6.4	11.5	1.1	3.9	0.9	3.0	1.9	1.5
31	Smoke	12.7	1.9	5.2	7.9	1.2	2.9	0.7	2.4	1.0	1.0
32	Smoke	12.8	1.9	4.6	7.2	0.9	3.3	0.8	2.3	1.11	1.1
33	Smoke	13.7	2.0	5.2	6.1	1.4	4.2	1.0	3.0	0.8	1.4
34	Smoke	17.3	3.1	6.8	7.1	2.7	6.5	1.7	3.8	1.5	2.2
**Mean**	**Smoke**	**117.2**	**38.9**	**26.9**	**24.4**	**12.8**	**51.9**	**13.3**	**14.5**	**7.4**	**12.7**

Notes: Non-smoke-exposed samples were randomly selected from Oregon State University research vintages made between 2013 and 2016, which were not barrel-aged**.**

Smoke: Smoke-exposed samples were obtained from grape growers and winemakers with a risk of smoke taint in the summer of 2020, the most destructive wildfire season on record in Oregon.

Table S3. Thirty-five tentative annotated glycosides (level 2 annotations) identified from non-smoke-exposed and smoke-exposed wines.

No.	Accepted Compounds	Adducts detected	Formula	Exact mass	Mass Error (mg/L, ppm)
1	Syringyl-β-D-Glucopyranoside-Iso	$[M+H]^+$, $[M+NH_4]^+$, $[M+Na]^+$	$C_{14}H_{20}O_8$	316.1158	-3.14
2	Syringyl-β-D-Glucopyranoside	$[M+NH_4]^+$, $[M+Na]^+$	$C_{14}H_{20}O_8$	316.1158	-2.24
3	Deoxyhexose-H-Cresol	$[M+NH_4]^+$, $[M+Na]^+$	$C_{19}H_{28}O_{10}$	416.1682	-3.50
4	Deoxyhexose-H-Cresol-Iso	$[M+Na]^+$	$C_{19}H_{28}O_{10}$	416.1682	-3.69
5	Deoxyhexose-H-P-Phenol	$[M+Na]^+$	$C_{23}H_{34}O_{14}$	534.1948	-4.10
6	Deoxyhexose-P-4-Ethylguaiacol	$[M+Na]^+$	$C_{20}H_{30}O_{10}$	430.1839	-1.93
7	Guaiacyl-β-D-Gentiobioside-Iso1	$[M+NH_4]^+$	$C_{19}H_{28}O_{12}$	448.1581	-4.50
8	Guaiacyl-β-D-Gentiobioside	$[M+Na]^+$	$C_{19}H_{28}O_{12}$	448.1581	-4.12
9	Guaiacyl-β-D-Gentiobioside-Iso2	$[M+Na]^+$	$C_{19}H_{28}O_{12}$	448.1581	-4.11
10	Guaiacyl-β-D-Gentiobioside-Iso3	$[M+Na]^+$	$C_{19}H_{28}O_{12}$	448.1581	7.61
11	H-4-Methylguaiacol	$[M+NH_4]^+$	$C_{14}H_{20}O_7$	300.1209	-3.68
12	H-4-Methylguaiacol-Iso2	$[M+H]^+$	$C_{14}H_{20}O_7$	300.1209	-3.18
13	H-4-Methylsyringol	$[M+Na]^+$	$C_{15}H_{22}O_8$	330.1315	-1.53
14	H-Guaiacol	$[M+Na]^+$	$C_{13}H_{18}O_7$	286.1052	-2.00
15	H-Guaiacol-Iso2	$[M+Na]^+$	$C_{13}H_{18}O_7$	286.1052	-3.35
16	H-Guaiacol-Iso3	$[M+Na]^+$	$C_{13}H_{18}O_7$	286.1052	-3.56
17	H-H-P-4-Ethylguaiacol-Iso1	$[M+H-H_2O]^+$	$C_{24}H_{36}O_{14}$	548.2105	-7.46

18	H-H-P-4-Ethylguaiacol	$[M+H]^+$	$C_{24}H_{36}O_{14}$	548.2105	-7.68
19	H-H-P-4-Ethylguaiacol-Iso2	$[M+H-H_2O]^+$	$C_{24}H_{36}O_{14}$	548.2105	-9.13
20	Benzyl O-[arabinofuranosyl-(1->6)-glucoside]	$[M+NH_4]^+$	$C_{18}H_{26}O_{10}$	402.1526	-4.49
21	H-P-4-Methylguaiacol-Iso	$[M+Na]^+$	$C_{19}H_{28}O_{11}$	432.1632	-4.63
22	H-P-4-Methylguaiacol	$[M+H]^+$	$C_{19}H_{28}O_{11}$	432.1632	-7.97
23	H-P-Guaiacol	$[M+Na]^+$	$C_{18}H_{26}O_{11}$	418.1475	-7.79
24	H-P-Guaiacol-Iso1	$[M+Na]^+$	$C_{18}H_{26}O_{11}$	418.1475	-5.29
25	H-P-Guaiacol-Iso2	$[M+Na]^+$	$C_{18}H_{26}O_{11}$	418.1475	-3.63
26	H-P-Guaiacol-Iso3	$[M+Na]^+$	$C_{18}H_{26}O_{11}$	418.1475	-4.93
27	H-P-P-4-Methylguaiacol	$[M+Na]^+$	$C_{24}H_{36}O_{15}$	564.2054	-4.72
28	P-H-Cresol	$[M+H]^+$	$C_{18}H_{26}O_{10}$	402.1526	-6.84
29	P-H-Cresol-Iso2	$[M+H-H_2O]^+$	$C_{18}H_{26}O_{10}$	402.1526	-7.78
30	P-H-Cresol-Iso3	$[M+H-H_2O]^+$	$C_{18}H_{26}O_{10}$	402.1526	-9.25
31	P-H-Cresol-Iso4	$[M+H]^+$, $[M+NH_4]^+$, $[M+Na]^+$	$C_{18}H_{26}O_{10}$	402.1526	-3.61
32	P-P-H-Cresol	$[M+Na]^+$	$C_{23}H_{34}O_{14}$	534.1948	-2.80
33	Syringyl-β-D-Gentiobioside	$[M+NH_4]^+$	$C_{20}H_{30}O_{13}$	478.1686	-4.53
34	Syringyl-β-D-Gentiobioside-Iso1	$[M+H]^+$, $[M+Na]^+$	$C_{20}H_{30}O_{13}$	478.1686	-8.89
35	Syringyl-β-D-Gentiobioside-Iso2	$[M+H-H_2O]^+$	$C_{20}H_{30}O_{13}$	478.1686	-8.01

Note: H-Hexose; P-Pentose; Iso-Isomer

Table S4. Correlation matrix for VPs and VP-glycosides in non-smoke-exposed and smoke-exposed wines with Pearson's correlation analysis.

	4-methylguaiacol-free	*p*-cresol-free	guaiacol-total	guaiacol-free	*m*-cresol-free	4-methylguaiacol-total	*p*-cresol-total	*m*-cresol-total	*o*-cresol-total	*o*-cresol-free
Syringyl-β-D-Glucopyranoside-Iso	0.15	0.18	0.17	0.11	0.28	0.26	0.29	0.39	0.36	0.38
H-Guaiacol-Iso2	0.15	0.23	0.26	0.17	0.25	0.28	0.34	0.35	0.39	0.39
P-H-Cresol-Iso2	0.16	0.18	0.23	0.15	0.29	0.28	0.32	0.38	0.39	0.38
H-4-Methylguaiacol-Iso2	0.19	0.23	0.25	0.19	0.38	0.30	0.32	0.41	0.42	0.46
H-P-4-Methylguaiacol-Iso	0.22	0.33	0.25	0.19	0.37	0.32	0.42	0.46	0.44	0.46
Syringyl-β-D-Gentiobioside-Iso2	0.14	0.22	0.28	0.21	0.28	0.25	0.32	0.25	0.36	0.36
Deoxyhexose-H-Cresol	0.20	0.26	0.32	0.24	0.31	0.34	0.36	0.45	0.43	0.42
Guaiacyl-β-D-Gentiobioside-Iso3	0.19	0.21	0.32	0.25	0.36	0.33	0.32	0.44	0.41	0.42
P-H-Cresol-Iso4	0.20	0.27	0.23	0.18	0.28	0.31	0.32	0.42	0.37	0.35
Deoxyhexose-P-4-Ethylguaiacol	0.34	0.29	0.29	0.26	0.38	0.44	0.40	0.54	0.50	0.53
H-H-P-4-Ethylguaiacol-Iso1	0.23	0.30	0.31	0.24	0.30	0.35	0.36	0.40	0.45	0.47
H-H-P-4-Ethylguaiacol	0.19	0.23	0.26	0.18	0.31	0.31	0.32	0.39	0.42	0.43

P-H-Cresol-Iso3	0.17	0.10	0.08	0.05	0.26	0.22	0.21	0.35	0.28	0.31
Syringyl-β-D-Gentiobioside-Iso1	0.11	0.19	0.15	0.10	0.28	0.21	0.26	0.29	0.35	0.39
H-4-Methylsyringol	0.17	0.09	0.24	0.17	0.27	0.27	0.22	0.31	0.35	0.35
H-Guaiacol	0.29	0.19	0.27	0.24	0.36	0.35	0.30	0.40	0.42	0.45
H-Guaiacol-Iso3	0.32	0.43	0.28	0.27	0.45	0.38	0.41	0.46	0.46	0.50
H-P-P-4-Methylguaiacol	0.28	0.36	0.26	0.23	0.39	0.33	0.40	0.41	0.39	0.38
Syringyl-β-D-Gentiobioside	0.28	0.31	0.24	0.22	0.42	0.34	0.31	0.44	0.42	0.45
Syringyl-β-D-Glucopyranoside	0.21	0.40	0.33	0.27	0.28	0.30	0.42	0.28	0.41	0.36
H-4-Methylguaiacol	0.19	0.27	0.29	0.23	0.24	0.32	0.31	0.41	0.41	0.39
H-H-P-4-Ethylguaiacol-Iso2	0.23	0.23	0.33	0.26	0.18	0.33	0.31	0.27	0.40	0.41
P-P-H-Cresol	0.40	0.31	0.41	0.38	0.36	0.43	0.39	0.43	0.47	0.48
H-P-Guaiacol	0.75	0.77	0.78	0.77	0.80	0.82	0.77	0.79	0.86	0.85
H-P-Guaiacol-Iso1	0.58	0.68	0.71	0.65	0.63	0.70	0.71	0.74	0.76	0.74
Deoxyhexose-H-Cresol-Iso	0.35	0.39	0.41	0.37	0.49	0.45	0.49	0.51	0.56	0.61
P-H-Cresol	0.41	0.38	0.41	0.36	0.49	0.49	0.49	0.57	0.56	0.59
Guaiacyl-β-D-Gentiobioside	0.37	0.35	0.31	0.32	0.48	0.41	0.44	0.51	0.47	0.53
H-P-4-Methylguaiacol	0.22	0.45	0.48	0.41	0.46	0.38	0.42	0.37	0.55	0.58
Guaiacyl-β-D-Gentiobioside-Iso1	0.42	0.58	0.57	0.54	0.52	0.51	0.52	0.45	0.58	0.61

H-P-Guaiacol-Iso3	0.33	0.23	0.10	0.17	0.26	0.21	0.27	0.22	0.20	0.26
Guaiacyl-β-D-Gentiobioside-Iso2	0.51	0.54	0.44	0.45	0.57	0.51	0.54	0.51	0.55	0.58
H-P-Guaiacol-Iso2	0.40	0.49	0.33	0.35	0.44	0.40	0.47	0.45	0.43	0.43
Deoxyhexose-H-P-Phenol	0.22	0.30	0.13	0.17	0.39	0.19	0.24	0.22	0.28	0.38
Benzyl O-[arabinofuranosyl-(1->6)-glucoside]	0.20	0.45	0.37	0.36	0.41	0.33	0.30	0.33	0.45	0.54

Note: H-Hexose; P-Pentose; Iso-Isom.
